# The Association of Lower Levels of Baseline Proteinuria With Earlier Remission in Primary Membranous Nephropathy

**DOI:** 10.7759/cureus.61918

**Published:** 2024-06-07

**Authors:** Jerry Joseph, Thirumavalavan Subramanian, Murugesan Vellaisamy, Srinivasaprasad ND, Sujith Surendran, Thirumalvalavan Kaliaperumal, Poongodi Annadurai, Nived Haridas, Edwin Fernando

**Affiliations:** 1 Nephrology, Government Stanley Medical College and Hospital, Chennai, IND

**Keywords:** secondary membranous nephropathy, pla2r, nephrotic syndrome, membranous nephropathy, primary membranous nephropathy

## Abstract

Aim

To study the clinical profile and course and to assess the outcome of patients with biopsy-proven primary membranous nephropathy (MN).

Methods

This study was carried out in a tertiary care hospital between December 2017 and December 2021 on four-year retrospective biopsy-proven patients with membranous nephropathy (MN). Urinary proteins, serum albumin, and serum creatinine were the baseline investigations that were performed. Special tests were done whenever necessary. Patients were treated with a modified Ponticelli (MP) regimen whenever needed. Patients were followed up after treatment administration for a minimum of a year.

Results

The study was done in 48 biopsy-proven MN patients. Thirty-six patients had primary MN with a mean age of 47+/-11.7 years. The male-female ratio was 2.6:1. Hypertension was present in 39% (14 patients), microscopic hematuria in 28% (10 patients), and acute kidney injury in 22% (8 patients). The mean 24-hour urinary protein was 11.2+/-2.9 g/day. PLA2R was positive in 78% (28 patients) of primary MN patients. Spontaneous remission was noted in 13.8% (5 patients) who were treated conservatively. Spontaneous remission was associated with lower baseline proteinuria (p<0.001), higher baseline serum albumin (p*<*0.001), and PLA2R negativity (p=0.04). Complete or partial treatment response was noted in 74.2% (23 patients). Treatment remission was associated with lower baseline proteinuria (p*=*0.018).

Secondary membranous nephropathy (secondary MN) was diagnosed in 12 patients. Eleven were class V lupus nephritis, all women, and one male person living with HIV/AIDS (PLHA).

Conclusions

The majority of our primary MN patients were PLA2R positive on renal biopsy. Statistically significant factors associated with spontaneous remission were lower proteinuria, higher serum albumin at baseline, and PLA2R negativity. Treatment response was associated with lower proteinuria at presentation. The most common cause of secondary MN was lupus nephritis.

## Introduction

Membranous nephropathy is one of the leading causes of nephrotic syndrome in adults [1,2]. Eighty percent of individuals have no known etiology for their MN (primary MN); the other cases are associated with drugs or diseases like malignancies, hepatitis virus infection, or systemic lupus erythematosus [2]. Primary MN is an autoimmune disease wherein circulating antibodies target glomerular podocytic antigens. Nephrotic syndrome is the most common presentation of primary MN patients, with sub-nephrotic proteinuria accounting for the remainder [1].

A paradigm shift in disease diagnosis and monitoring was brought about by the 2009 identification of phospholipase A2 receptor (PLA2R) as the primary antigen in adults. Since then, several additional antigens have been identified [2]. The course of the disease is unpredictable; around one-third of patients will have spontaneous remission. Immunosuppressive therapy with corticosteroids and cyclophosphamide (MP regimen) has significantly decreased the need for renal replacement therapy in patients with a high risk of progression [2]. Other treatments (rituximab and calcineurin inhibitors) have been developed due to adverse effects like carcinogenic risks and infections of the modified Ponticelli (MP) regimen. However, when calcineurin inhibitors are discontinued, disease relapses are common [2]. Physicians frequently have to treat primary MN with an MP regimen since rituximab therapy is still more expensive than other medications, especially in places with limited resources. This study aims to determine the clinical profile, course, and outcome of biopsy-proven primary MN.

## Materials and methods

This study was conducted in the Department of Nephrology in a tertiary care government hospital. The Institutional Ethics Committee of Government Stanley Medical College and Hospital approved the study, with the approval number ECR/131/Inst/TN/2013/RR-22/20220506.

The study population included four years of retrospective renal biopsy-proven patients with MN on regular follow-up in the Nephrology Outpatient Department between December 2017 and December 2021. Inclusion in the study was limited to patients who had at least a year of follow-up. Patients with irregular follow-up or those who defaulted treatments were excluded.

Their details were collected from registers and the Department of Nephrology outpatient records. Their presentation, past medical and drug histories, examination details, laboratory investigations, biopsy reports, treatment, course, and outcome were all collected and analyzed.

Laboratory investigations included basic investigations like urine routine examination, 24-hour urine proteins, hemogram, blood urea, serum creatinine, serum albumin, serum proteins, serum cholesterol, and serum electrolytes. Light microscopy and immunofluorescence staining for IgG, IgM, IgA, kappa, lambda, and PLA2R were done in all renal biopsies. Special investigations like anti-nuclear antibodies, human immunodeficiency virus, hepatitis-B Ag, and antibody to hepatitis C virus were done when renal biopsy is PLA2R negative.

Patients with an underlying cause for MN were diagnosed as secondary MN and those without were diagnosed as primary MN.

The hospital’s established treatment plan for patients with primary MN was based on Kidney Disease Improving Global Outcomes (KDIGO) guidelines, and that was followed for the treatment of our patients. Patients with massive proteinuria or complications like acute kidney injury or thromboembolism were treated with an MP regimen at the presentation time. Others were conservatively managed with maximum tolerable doses of angiotensin-converting enzyme inhibitors (ACEi), initially for three to six months, and those who did not achieve remission or whose proteinuria worsened were started on an MP regimen. Patients were monitored for six months following the MP regimen for the remission of the disease. If they did not achieve remission, they were considered as resistant to the first dose of the MP regimen and were readministered a second dose after assessing the risk/benefit of a further dose of cyclophosphamide.

The MP regimen included three days of pulse intravenous methylprednisolone (1 g/day) followed by 27 days of oral prednisolone (0.5 mg/kg/day) in months one, three, and five, and only oral cyclophosphamide (2 mg/kg) in months two, four and six.

The criteria used to define remission and relapse were as follows. 1) Complete remission: proteinuria reduction to <0.30 g/day; 2) partial remission: >50% reduction of proteinuria from baseline to between 0.3 and 3.5 g/day; 3) relapse: proteinuria recurrence of >3.5g/day after complete or partial remission.

Statistical analysis

All statistical analyses were done using IBM SPSS Statistics for Windows, Version 20 (Released 2011; IBM Corp., Armonk, New York, United States). The statistical analysis consisted of calculating means and proportions. We used appropriate significance tests such as the chi-square test and the Student’s t-test to test the association between spontaneous or treatment remission of MN with various patient characteristics.

## Results

**Figure 1:**
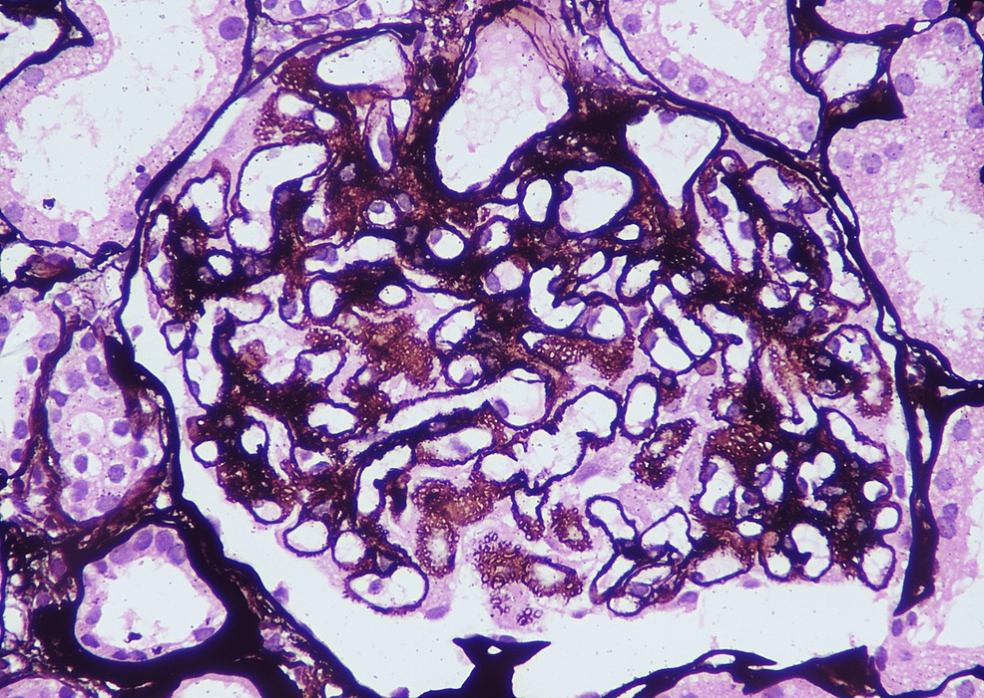
Light microscopy in MN. Glomeruli in advanced MN, showing spikes and pinhole pattern (Jones methenamine silver stain). MN: membranous nephropathy

**Figure 2:**
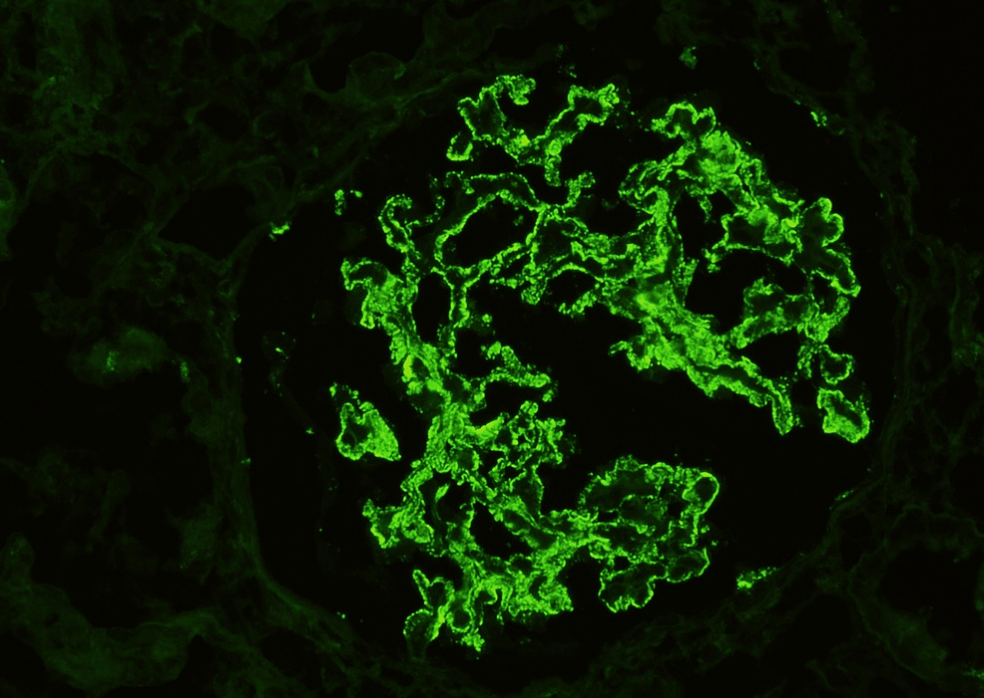
Immunofluorescence in MN. Glomerulus with a diffuse, finely granular deposit of immunoglobulin G along the outer surface of capillary walls. MN: membranous nephropathy

We had 48 biopsy-proven MN patients (Figures 1, 2).

**Figure 3:**
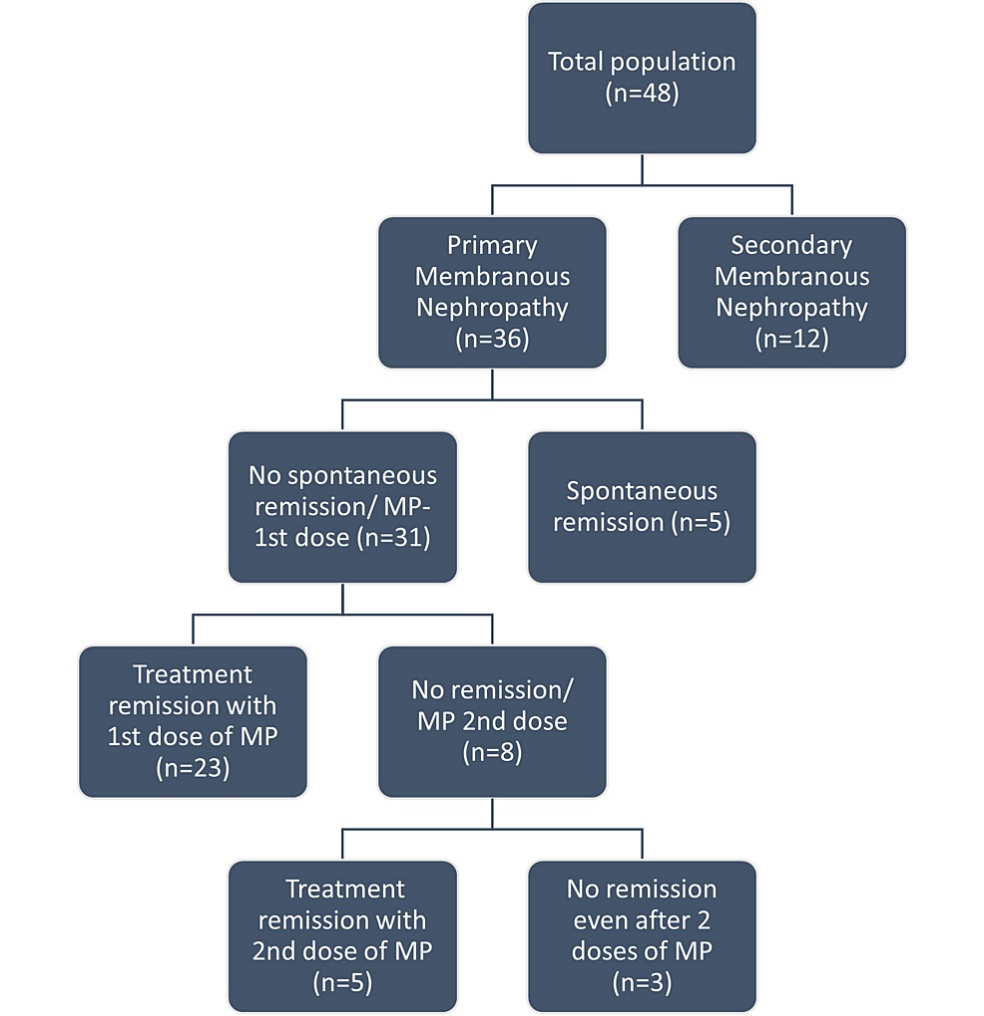
Flow diagram with the number of patients with different outcomes.

Thirty-six patients (75%) had primary MN (Figure 3). Among these, 26 (72.3%) were male, and 10 (27.7%) were female. The majority of patients with primary MN were in their 4th-5th decade, constituting 72% (n=26) of patients, with a mean age of 47+/-11.7 years. The male-to-female ratio was 2.6:1. Thirty-nine percent (n=14) had hypertension, with 41.3% (12 of 29 patients) of patients over 40 years having hypertension compared to only 28.5% (two of seven patients) below 40 years. In the study group, microscopic hematuria was observed in 28% (n=10) of cases. Edema was a presenting feature of 97% (n=35) of patients. Oliguria was present in 19.5% (n=7) of patients. Venous thromboembolism was present in 11% (n=4). PLA2R in biopsy specimens was positive in 78% (n=28) of the patients.

The cases' baseline characteristics were proteinuria of 11.2+/-2.9 gm/day, serum albumin of 2.2+/-0.3 g/dL, and serum creatine of 1.5+/-1.5 mg/dL (Table 1). The incidence of individuals with or without renal impairment was established by taking the arbitrary serum creatinine value of 1.2 mg/dl as the threshold for renal dysfunction. Renal dysfunction was observed in 22% (n=8). On renal biopsy, four (11%) had acute interstitial nephritis (AIN), two (5.5%) had acute tubular injury (ATI), and two (5.5%) had combined AIN and ATI.

**Table 1:** Baseline characteristics of primary MN patients (n=36). The data has been represented as number (n), percentage (%), and mean ± standard deviation (SD). MN: membranous nephropathy; PLA2R: phospholipase A2 receptor

Clinical parameters	n	Percentage (%)
Age, years (mean ± SD)	47 ± 11.7	NA
Male	26	62.3
Female	10	27.7
Hypertension	14	38.8
Edema	35	97
Oliguria	7	19.5
Microscopic hematuria	10	28
Venous thromboembolism	4	11.1
Nephrotic syndrome	35	97
Renal dysfunction	8	22
Serum creatinine, mg/dl (mean ± SD)	1.5 ± 1.5	NA
Serum albumin, g/dl (mean ± SD)	2.2 ± 0.3	NA
Proteinuria, g/24 hours (mean ± SD)	11.2 ± 2.9	NA
Serum cholesterol, mg/dl (mean ± SD)	329 ± 29	NA
PLA2R positive	28	78

Of 36 patients with primary MN, 40% (n=14) were started on an MP regimen at the time of detection due to massive proteinuria or other complications like acute kidney injury or venous thromboembolism. Rest (22 patients (60%)) were initially managed conservatively with antiproteinuric measures. Among those managed conservatively, five patients (22.7%) achieved spontaneous remission within six months, and the rest (17 patients (77.2%)) were started on an MP regimen. We observed that patients who developed spontaneous remission had significantly lower baseline proteinuria (p<0.001), higher baseline serum albumin (p<0.001), and PLA2R negativity in biopsy specimens (p=0.04) than those who did not have spontaneous remission. Two out of five patients (40%) with spontaneous remission had PLA2R positivity in the biopsy specimen compared to 24 out of 31 patients (77%) without spontaneous remission (Table 2).

**Table 2:** Factors associated with spontaneous remission. The data has been represented as mean ± standard deviation (SD) and percentage (%). PLA2R: phospholipase A2 receptor

Spontaneous remission	Yes (n=5)	No (n=17)	p
Proteinuria, g/24 hours (mean ± SD)	6.8 ± 3.1	10.9 ± 4.5	<0.001
Serum albumin, g/dl (mean ± SD)	2.8 ± 0.6	2.3 ± 0.5	<0.001
PLA2R positive in biopsy specimen (%)	40	77	0.04

Of the 31 patients treated with the MP regimen, 23 (74.2%) achieved partial or complete remission. Patients who achieved partial or complete remission to the MP regimen had significantly lower baseline proteinuria than those who did not respond to therapy (p=0.018). Remission to the MP regimen is not associated with serum albumin (p=0.91) or PLA2R positivity (p=1.1). Twenty out of the 23 patients (87%) with treatment remission had PLA2R positivity on biopsy specimens compared to 6 out of the 8 patients (75%) who were treatment-resistant (Table 3).

**Table 3:** Factors associated with treatment remission. The data has been represented as mean ± standard deviation (SD) and percentage (%). PLA2R: phospholipase A2 receptor

Treatment remission	Yes (n=23)	No (n=8)	p
Proteinuria, g/24 hours (mean ± SD)	11 ± 1.4	13.6 ± 2.7	0.018
Serum albumin, g/dl (mean ± SD)	2.3 ± 0.6	2.1 ± 0.4	0.91
PLA2R positive in biopsy specimen (%)	87	75	1.1

Nineteen percent (n=6) had a relapse during follow-up. Of the patients who achieved remission, we found no statistically significant difference in baseline proteinuria between relapsers and non-relapsers (Table 4).

**Table 4:** Factors associated with disease relapse. The data has been represented as mean ± standard deviation (SD).

Relapse	Yes (n=6)	No (n=27)	p
Proteinuria, g/24 hours (mean ± SD)	11.5 ± 1.9	10.7 ± 1.3	0.71

Eight patients (26%) were resistant even after six months of treatment with the MP regimen. They were given a second course of the MP regimen six months after finishing the first course of treatment. Five patients (62.5%) attained remission (Table 5).

**Table 5:** Treatment response to first and second doses of MP. The data has been represented as a number (n). MP: modified Ponticelli

Treatment response	Remission	No remission
Response to 1st dose of MP, n	23	8
Response to 2nd dose of MP, n	5	3

One patient (3.2%) developed pneumonia, but it was not life-threatening. Two patients (6.4%) developed urinary tract infections and two patients (6.4%) developed neutropenia (Table 6).

**Table 6:** Treatment-related adverse effects. The data has been represented as number (n) and percentage (%).

Adverse effects	n	Percentage (%)
Urinary tract infection	2	6.4
Neutropenia	2	6.4
Pneumonia	1	3.2

Secondary MN was diagnosed in 12 patients (25%). Eleven (91.6%) were due to class V lupus nephritis; all were women. There was one male patient with people living with HIV/AIDS (PLHA) (Table 7). All the patients had non-nephrotic proteinuria. All the lupus nephritis patients were treated conservatively with anti-proteinuric measures and the PLHA patient was treated with highly active antiretroviral therapy (HAART).

**Table 7:** Etiology of secondary MN (n=12). The data has been represented as number (n) and percentage (%). MN: membranous nephropathy; PLHA: people living with HIV/AIDS

Etiology of secondary MN	n	Percentage (%)
Lupus nephritis	11	91.6
PLHA	1	8.4

## Discussion

One of the most common causes of adult nephrotic syndrome is MN. During the study period of four years, out of 756 native kidney biopsies done in our institute, 12% were of MN. In our study, the highest proportion of patients with primary MN was in the 4th-5th decade, constituting 72% (n=26) with primary MN, and the mean age of patients was 47+/-11.7 years. This agrees with the established literature in MN, where in multiple studies, it is shown that MN is commonly seen in adults over the age of 40 years [1,3]. There is a male predilection for primary MN; in our study, males are 2.6 times more commonly affected than females. Similar sex predilections are seen in other studies [1,4,5].

The baseline proteinuria in our patients with primary MN is 11.2+/-2.9 g/day. It is high compared to studies from North India, Europe, and Pakistan of 6 g/day, 7.1 g/day, and 7.2 g/day, similar to a study by Hemanth Kumar et al. of 10.5 g/day [1]. All except one patient with primary MN had nephrotic syndrome at presentation. Most of our secondary MN patients presented with sub-nephrotic proteinuria.

Thirty-nine percent (n=14) of our patients had hypertension, and 28% (n=10) had microscopic hematuria at presentation. While certain studies found a similar incidence of microscopic hematuria and hypertension, other studies revealed a higher incidence of the latter [1].

Among different nephrotic syndromes, patients with MN have the highest propensity to develop thromboembolic complications, mainly renal vein thrombosis. Four (11.1%) of our patients with primary MN had some form of venous thromboembolism at presentation.

As shown by Troyanov et al. [6] in their study, hypertension and reduced GFR are more common at presentation in older patients, and this may reflect tubulointerstitial and vascular changes on biopsy independent of the severity of MN. Eight (22%) of our primary MN had renal dysfunction at presentation. Four patients (11%) on renal biopsy had AIN, two (5.5%) had ATI, and two (5.5%) had combined AIN and ATI. None of the patients had chronic changes of MN. Renal function in all these patients improved on follow-up.

Most (78% (n=28)) of our primary MN patients were PLA2R positive on renal biopsy. Similar rates of PLA2R positivity are seen in other studies [7,8]. In the study conducted in South India by Kurien et al. [9], 35% of patients with MN have a history of traditional indigenous medicine use, and most were NELL1 positive in renal biopsy. We haven’t tested for NELL1, as it was unavailable in our study period.

We observed that patients who developed spontaneous remission had significantly lower baseline proteinuria (p<0.001), higher baseline serum albumin (p<0.001), and PLA2R negativity (p=0.04) than those who did not have spontaneous remission. Proteinuria of patients with and without spontaneous remission were 6.8+/-3.1 and 10.9+/-4.5, respectively. Among patients with and without spontaneous remission, serum albumin was 2.8+/-0.6 and 2.3+/-0.5, respectively. It is well described in the literature that spontaneous remission occurs in up to 30% of patients with MN. Female gender and lower-grade proteinuria at presentation are the only two features associated with a higher likelihood of spontaneous remission [10]. The degree of proteinuria is a consistently reported factor associated with complete or partial remission. Some investigators have found that the amount of proteinuria at presentation could predict the outcome of MN [11]. Cattran et al. designed a model for the risk of progression in primary MN based on the severity of proteinuria over a six-month observation period [12]. This model incorporated many studies of Finnish, Italian, and Canadian patients. Based on these studies, Cattaran et al. reported that patients with normal baseline serum creatinine and proteinuria of less than 4 g/day over six months can be considered low risk compared to patients with normal serum creatinine and proteinuria of 4 to 8 g/day. Furthermore, patients with abnormal serum creatinine and proteinuria of more than 8 g/day carry a high risk of progression.

Treatment with a combination of steroid and cyclophosphamide (MP regimen) remains the time-tested modality in patients with moderate and high risk of progression. Rituximab is gaining popularity nowadays due to its lesser adverse effects, but we haven't tried it on our patients because of logistic issues. Of 31 patients (86%) treated with the MP regimen, 23 (74.2%) achieved partial or complete remission. This is like other studies from India. Jha et al. reported a remission rate of 72% [13], and Ram et al. reported a remission rate of 77.5% [14]. Patients who achieved partial or complete remission to the MP regimen had significantly lower proteinuria than those who did not respond to therapy (p=0.018). Proteinuria of patients with and without treatment response was 11+/-1.4 and 13.6+/-2.7, respectively. Treatment remission was not associated with baseline serum albumin or PLA2R positivity.

Of the patients who had any form of remission (spontaneous or treatment), we observed a relapse rate of 19% (n=6) during follow-up. Of the patients who achieved remission, we found no statistically significant difference in baseline proteinuria between relapsers and non-relapsers. Ramachandran et al. reported a relapse rate of 6.7% [15]. A relapse rate of 30% was observed by Eriguchi et al. [16] from Japan and 23% by Jha et al. from India.

Treatment with the potent immunosuppressive regimen of MP was not without side effects. One patient (3.2%) developed pneumonia, but it was not life-threatening. Two patients (6.4%) developed urinary tract infections. Two patients (6.4%) developed neutropenia.

Our study has several limitations. First, the generalizability of our findings may be limited as the study was conducted in only one center with a small population. Second, a prospective study would give better results compared to a retrospective study. Third, we haven’t used serum anti-PLA2R antibody levels for prognostication of our primary MN patients, which has become the standard in many parts of the world. Fourth, all our primary MN patients requiring immunosuppression were treated with an MP regimen. Less toxic and efficacious therapy like rituximab is available for managing MN. However, due to logistic reasons, we were unable to use rituximab. Fifth, since the description of PLA2R, many newer antigens like NELL1 have been described in MN. We haven’t checked for the positivity of these antigens.

## Conclusions

The majority of our primary MN patients were PLA2R positive on renal biopsy. The most common presentation in patients with primary MN was nephrotic syndrome, while hypertension and microscopic hematuria were also frequently observed. Key factors associated with the spontaneous remission of primary MN were lower baseline proteinuria, higher baseline serum albumin levels, and PLA2R negativity. A lower level of protinuria at presentation was associated with a better treatment response. Lupus nephritis was the most frequent cause of secondary MN, typically presenting with non-nephrotic range proteinuria.

## References

[1] Hemanth Kumar MK, Sandhu J, Sandhu JS (2022). Profile and primary treatment outcomes in membranous nephropathy. Saudi Med J.

[2] Ronco P, Beck L, Debiec H (2021). Membranous nephropathy. Nat Rev Dis Primers.

[3] Jiang Z, Cai M, Dong B (2018). Clinicopathological features of atypical membranous nephropathy with unknown etiology in adult Chinese patients. Medicine (Baltimore).

[4] Ronco P, Debiec H (2015). Membranous nephropathy: a fairy tale for immunopathologists, nephrologists and patients. Mol Immunol.

[5] Couser WG (2017). Primary membranous nephropathy. Clin J Am Soc Nephrol.

[6] Troyanov S, Roasio L, Pandes M, Herzenberg AM, Cattran DC (2006). Renal pathology in idiopathic membranous nephropathy: a new perspective. Kidney Int.

[7] Francis JM, Beck LH Jr, Salant DJ (2016). Membranous nephropathy: a journey from bench to bedside. Am J Kidney Dis.

[8] Ronco P, Debiec H (2015). Pathophysiological advances in membranous nephropathy: time for a shift in patient’s care. Lancet.

[9] Kurien AA, Prema Ks J, Walker PD, Caza TN (2022). Traditional indigenous medicines are an etiologic consideration for NELL1-positive membranous nephropathy. Kidney Int.

[10] Hladunewich MA, Troyanov S, Calafati J, Cattran DC (2009). The natural history of the non-nephrotic membranous nephropathy patient. Clin J Am Soc Nephrol.

[11] Pei Y, Cattran D, Greenwood C (1992). Predicting chronic renal insufficiency in idiopathic membranous glomerulonephritis. Kidney Int.

[12] Cattran DC, Pei Y, Greenwood CM, Ponticelli C, Passerini P, Honkanen E (1997). Validation of a predictive model of idiopathic membranous nephropathy: its clinical and research implications. Kidney Int.

[13] Jha V, Ganguli A, Saha TK (2007). A randomized, controlled trial of steroids and cyclophosphamide in adults with nephrotic syndrome caused by idiopathic membranous nephropathy. J Am Soc Nephrol.

[14] Ram R, Guditi S, Kaligotla Venkata D (2015). A 10-year follow-up of idiopathic membranous nephropathy patients on steroids and cyclophosphamide: a case series. Ren Fail.

[15] Ramachandran R, Yadav AK, Kumar V (2017). Two-year follow-up study of membranous nephropathy treated with tacrolimus and corticosteroids versus cyclical corticosteroids and cyclophosphamide. Kidney Int Rep.

[16] Eriguchi M, Oka H, Mizobuchi T, Kamimura T, Sugawara K, Harada A (2009). Long-term outcomes of idiopathic membranous nephropathy in Japanese patients treated with low-dose cyclophosphamide and prednisolone. Nephrol Dial Transplant.

